# Effect of the anodal transcranial direct current electrical stimulation on cognition of medical residents with acute sleep deprivation

**DOI:** 10.5935/1984-0063.20220007

**Published:** 2022

**Authors:** Daniel San-Juan, Raúl Nathanael May Mas, Cuauhtémoc Gutiérrez, Jorge Morales, Ana Díaz, Gerardo Quiñones, Axel Kevin Galindo, Luis Armando Baigts, Cecilia Ximenez-Camilli, David Anschel

**Affiliations:** 1 Instituto Nacional de Neurología y Neurocirugía Manuel Velasco Suárez, Epilepsy Clinic - Mexico City - Mexico City - Mexico.; 2 Instituto Nacional de Neurología y Neurocirugía Manuel Velasco Suárez, Neurology Department - Mexico City - Mexico City - Mexico.; 3 Facultad de Estudios Superiores Iztacala, Psychology Posgraduate Department - Mexico City - Mexico City - Mexico.; 4 Instituto Nacional de Neurología y Neurocirugía Manuel Velasco Suárez, Unit of Cognition and Behavior - Mexico City - Mexico City - Mexico.; 5 New York University Comprehensive Epilepsy Center, Epilepsy Center - New York - New York - United States.

**Keywords:** Transcranial Direct Current Stimulation, Sleep Deprivation, Cognitive Remediation

## Abstract

**Background:**

Medical residents must sustain acute sleep deprivation, which can lead to nonfatal and fatal consequences in hospitals due to cognitive decline. Anodal transcranial direct current stimulation (a-tDCS) is a safe noninvasive neuromodulation technique that can induce depolarization of neurons. Previous studies in pilots have shown benefits against fatigue increasing wakefulness and cognitive performance. However, the effects of a-tDCS on cognition in acute sleep deprived healthcare workers remains unknown.

**Purpose:**

To evaluate cognitive changes in sleep deprived medical residents after one session of a-tDCS.

**Methods:**

Open clinical test-re-test study including 13 medical residents with acute sleep deprivation. Subjects received 1 session of bifrontal a-tDCS (2mAx20min), anodal over the left dorsolateral prefrontal region. Pre-and-post treatment subjects were tested with Beck anxiety inventory, Beck depression and HVLT tests, Rey´s and Taylor´s figures, Trail Making A/B, Stroop, Aleatory Digit retention test (WAIS), Digits and symbols and MoCA tests. Post-intervention was added the Executive functions and Frontal Lobes Neuropsychological Battery (BANFE2) test and changing the Taylor figure for Reyfigure.

**Results:**

Twelve medical residents were analyzed; 8 men and 4 women, 29.5 (+/-2.2) years mean age. All had a mean of 21.6 (+/-1.3) hours of sleep deprivation. There were no serious adverse events. We found statistically significant difference in Rey´s/Taylor´s figures (p=0.002), Trail Making Test (p=0.005), WAIS IV symbols (p=0.003), Word Stroop (p=0.021). BANFE-2 showed that the main affected area was the orbito-medial prefrontal region.

**Conclusion:**

a-tDCS appears safe and improves working memory, attention, response time and distractors elimination in acute sleep deprived medical residents.

## 1. INTRODUCTION

Long working hours and high cognitive demand may lead to acute and chronic sleep deprivation in professional guards, drivers, military personnel, and healthcare workers, among others. Sleep deprivation increases response time to stimuli and lowers precision, as well as causing inattention, emotional lability, somnolence, and chronic fatigue^1^. A previous study has shown that a state of acute sleep deprivation (17-19 hours) has an equivalent effect as a serum level of 0.05% of alcohol and the response speeds were up to 50% slower for some cognitive tests and accuracy measures considered in many countries as incompatible with safe driving^2^. A study in medical residents with 3 hours on average of sleep found a deterioration in reaction time^3^. Sleep disturbances and fatigue have been associated with an increased risk of developing depression and medical errors in healthcare workers^4^. Multiple cortical (bilateral intraparietal sulcus, bilateral insula, right prefrontal cortex, medial frontal cortex, and right parahippocampal gyrus) and subcortical structures including the hypothalamus have been implicated in sleep deprivation^5,6^.

Transcranial direct current stimulation (tDCS) is a noninvasive neuromodulation technique tested in patients with several neuro-psychiatric conditions and healthy volunteers^7^. Usually, tDCS involves the placement of 2 scalp electrodes on the head of the subject (cathode and anode). A low intensity electric current of 1-2 mA is applied over 20min. The flow of the electric current is distributed through the scalp to reach the cerebral cortex in a way that depending on the direction and intensity of the electric current, cortical excitability can be increased or decreased. In general, anodal tDCS (a-tDCS) increases cortical excitability through neuronal depolarization as cathodal tDCS generates hyperpolarization, causing cortical inhibition^7^. Anodal tDCS to the left dorsolateral prefrontal cortex (lDLPFC) has been tested in pilots and military personnel with chronic sleep deprivation and showed potential beneficial effects in the areas of cognition, professional performance, mood, and faster recovery of fatigue^8,9,10^. However, the effects of a-tDCS on cognition in acute sleep deprived healthcare workers remains unknown. The aim of this study was evaluate cognitive changes and safety in acutely sleep deprived medical residents after one session of a-tDCS.

## 2. MATERIAL AND METHODS

Using an open-label clinical study we included 13 adult medical residents with acute sleep deprivation (less than 5 hours of sleep in at least 24 hours) who were subject to one session of a-tDCS (2mA x 20min). This study was approved by the Research and Ethical local committees at the National Institute of Neurology and Neurosurgery in Mexico City; and conducted according to the Declaration of Helsinki and CONSORT guidelines.

Exclusion criteria were previous intake of any kind of stimulating substances prior to the a-tDCS, history of brain surgery or recent head trauma, regular use of any stimulant drugs, use of illegal substances, acute infectious diseases or any medical condition that could be exacerbated during the intervention, pregnancy or lactation, any metallic implant or device in the head or neck.

### 2.1. Neuropsychological evaluation:

The following validated tests were applied to all subjects in the study, 2 hours before a-tDCS session: Beck’s Anxiety Inventory, Beck’s Depression Test, Hopkins Verbal Learning Test, Rey´s Figure, Trail Making Test, FAS, Stroop, WAIS IV Digits and Symbols, Montreal Cognitive Assessment Test. Immediately after the experimental intervention we added the Executive Functions and Frontal Lobes Neuropsychological Battery-2 (BANFE-2 from Spanish) and changed the Rey´s Figure for Taylor´s Figures to avoiding the effects of learning. The neuropsychological tests were applied by two neuropsychologists trained in our Department.

We briefly describe each neuropsychological test applied to our participants.

#### 2.1.1. Montreal Cognitive Assessment Test (MoCA)

It is a brief screening instrument that allows evaluating different cognitive domains (attention, visoconstructive skills, memory, language, executive functions, calculation, and orientation). It evaluates 30 items, a score >26 points is considered normal performance. It has a Spanish version^11^.

#### 2.1.2. Hopkins Verbal Learning Test-Revised (HVLT)

This instrument evaluates verbal memory and learning. It includes 6 different lists of words to avoid the patient learning the instrument. It consists of 3 phases: learning curve, free evocation, and recognition. The first phase includes a list of 12 nouns from 4 different categories that are repeated 3 times to the evaluated person, who are asked to remember and repeat, thus obtaining a learning curve. After an interference of 20 to 25 minutes, the person is asked to freely evoke the list of words (free evocation phase). Immediately afterwards, 24 nouns are given, including the 12 words from the original list, 6 semantically related words and 6 unrelated words, from which the patient must identify which are the original words^12^ and has normative data standardized to the Mexican population^13^.

#### 2.1.3. Trail Making Test

This instrument evaluates executive functions, mainly: sustained attention, mental flexibility, inhibition and working memory. It consists of two parts: the first part consists of joining a sequence of numbers (1 to 25) that are distributed randomly along the page, in the shortest possible time. In the second part, the patient must alternate between numbers and letters (1-A, 2-B, 3-C, up to number 13), in the shortest possible time. It has been standardized for the Mexican population^13^.

#### 2.1.4. Verbal fluency test

In this test, the patient is asked to say the greatest number of words for one minute, without repeating them or using derivatives, and to start with a specific letter. To achieve this, executive control over other cognitive processes (such as attention, set change and self-monitoring) is required. Clinically, it has been related to the functioning of dorsolateral prefrontal regions of the brain^14^ and has standardized normative data for the Mexican population^15^.

#### 2.1.5. Stroop

The Stroop Color and Word Test assesses the ability to inhibit automated responses. It has three parts: in the first part, the names of colors must be read, in the second, the color of the graphic stimuli (100 elements “XXXX”) is said and in the third part, the word that names the color is different from the color of its impression, the person should say the color of the ink and avoid reading the word^14,16^. It has standardized normative data for the Mexican population^17^.

#### 2.1.6. Digit retention (Digit Span).

This instrument is part of the WAIS-IV battery^18^. It includes two parts: in the first part, the examinee is provided with series of numbers that will gradually increase and must repeat them in the same order, while in the second part they must repeat them in reverse order. These tasks assess attention volume and working memory.

#### 2.1.7. Digits and symbols

This instrument is part of WAIS-IV battery test^18^. The test consists of a matrix of digits that must be completed with the signs that correspond to each number in a time limit of two minutes. This scale assesses the speed of processing.

#### 2.1.8. Rey Complex Figure

This test is composed of a complex figure with no attributable meaning that must be copied and reproduced from memory. Its rating includes precision and location for each of the 18 elements that compose it. It evaluates processes related to perceptual organization and nonverbal memory processes^14^. and has normative data for the Mexican population^17^.

#### 2.1.9. Taylor Complex Figure

Taylor Complex Figure is similar to the Rey Complex Figure, and was designed for use in follow-up evaluations avoiding the effects of learning. It has been shown to be useful as a post-test measure^19^.

#### 2.1.10. Neuropsychological Battery of Executive Functions and Frontal Lobes (BANFE 2).

It consists of 15 subtests that integrate a complete profile of executive functioning, as well as the anatomicfunctional integrity of the orbito-frontal, medial dorsolateral and the anterior prefrontal cortices. The raw scores were normalized and sorted based in the severity of the changes and anatomic substrate of the findings. This was applied post-intervention to avoid the effects of learning.

#### 2.1.11. Beck´s test for depression.

In the form of a self-report, this instrument allows the assessment of the presence of depressive symptoms and to discriminate between mild, moderate, and severe depression symptoms^20^.

#### 2.1.12. Beck’s Anxiety Inventory.

Also, in self-report format, screens for the presence of anxiety symptoms. It allows to differentiate between minimal, mild, moderate and severe anxiety^21^.

Regarding the way in which they were applied, all the aforementioned tests were performed prior to tDCS stimulation, with the exception of the Taylor Complex Figure and the BANFE-2. Subsequently, the stimulation with a- tCDS was performed for 20 minutes, during which adverse effects were reported. Subsequently, all the previous tests were applied again, replacing the Rey Complex Figure with the Taylor Complex Figure, and adding the BANFE-2.

### 2.2 Intervention

A tDCS device® (Kowloon, Hong Kong) was used. A bi-frontal montage was applied; The anode was placed at F3 (EEG 10-20 system), and the cathode at F4. F3 overlies the left dorsolateral prefrontal cortex according to resting-state fMRI connectivity analysis^22^. A 5x5 cm (0.080 mA/cm^2^ at 2 mA) sponge soaked in saline was used with rubber electrodes. Stimulation was 2mA for 20 min. At the beginning of the stimulation, there was a ramp-up period of 10 seconds. If the skin resistance was within normal limits, the stimulation was provided continuously, and 2 seconds before the end of the stimulation session, the stimulator transitioned into the ramp-down phase lasting 10 seconds. Electrode resistance was constantly monitored (4 to 6 kΩ). All subjects were supervised to record any adverse event during and after the session. tDCS was performed by professionally trained personnel and the subjects remained seated comfortably.

The session, all patients underwent a repeat neuropsychological evaluation. All patients remained in observation 1 hour after the session to assess for adverse events and a questionnaire was used to evaluate for adverse effects based on Brunoni´s questionnaire for tDCS clinical trials^23^. Subjects were contacted 24 hours after the session for follow-up to ask about other potential adverse effects. All scores of tests before and after the session were compared in each participant.

### 2.3 Statistical analysis:

Data was processed using SPSS 23.0. Measures of central tendency and frequencies were used for descriptive statistics. Normality tests were used before the selection of non-parametric tests. The Wilcoxon signed-rank test was used to compare results before and after the tDCS session. A statistically significant value was taken if p=<0.05.

## 3. RESULTS

Thirteen medical residents were consented. Twelve were analyzed (one was excluded due to unfinished the pre-intervention neuropsychological evaluation). Table 1 details the socio-demographic characteristics of the participants; 8 men and 4 women, 29.5 (+/-2.2) years old mean age, all had a mean of 21.6 (+/-1.3) hours of sleep deprivation, 50% were internal medicine residents, 25% were neurology residents and the rest were from family medicine, among others.

**Table 1:** Socio-demographic characteristics of 12 medical residents.

Socio-demographic characteristic	n (%)
Gender	
Male	8 (67%)
Female	4 (34%)
Age (SD; range) years	29.5 (2.23, 21-30)
Medical specialty training	
Internal medicine	6 (50%)
Neurology	3 (25%)
Family medicine	1(18%)
Other	2(17%)
Sleep deprivation (SD; range) hours	21.6 (1.3; 5.7-6.9)

All medical residents except 1 were on call once every 96 hours, the other resident was on call once every 5 days. Table 2 shows the baseline and post-tDCS session changes in neuro-psychological tests. We found statistically significant differences in Rey´s/Taylor´s figures (p=0.002), Trail Making Test (p=0.005), WAIS IV symbols (p=0.003), Word Stroop (p=0.021). According to the BANFE-2 test the main brain region affected was orbito-medial (Table 3). Mild to moderate self-limiting and transient adverse events were reported in ten (83%) medical residents, however no serious adverse events were reported.

**Table 2:** Neuropsychological tests and their changes pre-and post- anodal tDCS.

Neuropsychological Test	Before	After	p
Beck Anxiety Inventory,Score	6.58	4.9	0.68
Beck Depression Inventory,Score	6.3	5.26	0.168
HVLT Learning,Score	25	26.6	0.129
HVLT Recall,Score	9.5	9.9	0.569
HVLT Right recall, Score	11.5	11.5	0.755
HVLT False related recall, Score	0.33	0.41	0.655
HVLT False non-related recall, Score	0	0	1.000
HVLT False total recall, Score	0.33	0.5	0.589
Rey-Osterrieth /Taylor´s complex figure test copy,Score	30	37.2	0.002
Rey-Osterrieth /Taylor´s complex figure, instantly,Score	24.7	43.3	0.212
Rey-Osterrieth /Taylor´s complex figure, delayed,Score	23.7	25.2	0.422
Trial Making A,Seconds	333	278	**0.200**
Trial Making B,Seconds	703	548	**0.005**
WAIS IV, Symbols and codes	81.7	93.4	**0.003**
WAIS IV, Progressive digital symbols	8.5	9.1	0.2929
WAIS IV, Regressive digital symbols	7.8	9.1	0.308
Stroop words	48.25	122	**0.021**
Stroop colors	79.5	84.3	0.102
Stroop words/colors	48.1	55.8	**0.028**
Stroop Interference	1.9	10	0.091
MoCa total, scores	28.3	27.5	0.666

**Table 3:** Executive functions and Frontal Lobes Neuropsychological Battery scores obtained by medical residents with acute sleep deprivation after a single a-tDCS session.

Parameter	Natural	Normalized	Classification
Subtotal orbito-medial	189 (+/- 33.8)	80	Mild to moderate changes
Subtotal anterior prefrontal	21.3 (+/- 40.8)	117	Normal high
Total dorsolateral	221 (+/- 17.6.8)	99	Normal
Total executive functions	436 (+/- 16.6.8)	99	Normal

### 3.1 Safety of tDCS

Ten (83%) medical residents reported at least one mild to moderate self-limiting and transient adverse event, with no need for pharmacological intervention. Six (20%) subjects reported a single adverse event, 2 (20%) subjects 2 events, and 2 (20%) subjects 3 events. Table 4 shows these adverse events and their frequencies. There were no cognitive changes reported for the participants in the 24 hours follow-up.

**Table 4:** Adverse events reported for medical residents after a-tDCS.

Adverse event	n(%)
Numbness	2 (20)
Headache	1 (10)
Nausea	2 (20)
Paresthesia	3 (30)
Somnolence	1 (10)
Pain	1 (10)

## 4. DISCUSSION

We found in this pilot study that one (20-min) session of a-tDCS over left dorsolateral prefrontal cortex was safe and had positive effects on cognitive performance of sleep deprived medical residents. Our results confirm that acute sleep deprivation (21.6 hours) in healthcare workers has deleterious effects on executive functions^3,4^ A previous prospective study that included Internal Medicine, Surgery and Ophthalmology residents with acute sleep deprivation from three third level hospitals located in Mexico City found that 81% of medical residents showed a detrimental effect in at least one of the cognitive tests and psychomotor skills^24^. Some of them could be reversed using a-tDCS to potentially diminished medical errors^25^ or sleep-related fatal vehicle accidents^26^.

Previous studies in military healthy volunteers with acute sleep deprivation using one session 20-min of a-tDCS over LDLPFC region induced enhancements in cognitive performance in arousal, working memory, attention and fatigue mitigation lasting up to 24 hours^8,9,10^. Our participants improved attention, response times and working memory. These findings are comparable to the effects induced by 400mg of caffeine in healthy acute sleep deprived subjects^10^. Other sleep studies in healthy subjects applying a-tDCS (0.26 mA/cm^2^) repeatedly (over 30 min) frontally (F3/F4) during deep Non-REM sleep improved declarative memory retention but not during wakefulness, this effect consisted of an acute increase in delta waves, accompanied by diminished power in the faster θ, lower α, and lower β EEG frequency bands during a-tDCS^27^. However, bi-frontal a-tDCS prior to sleep increases waking EEG gamma power and decreases total sleep time during the night^28^, this reduction in sleep deprived healthy subjects has been associated with a faster recovery from fatigue^8^.

The cognitive recovery using a-tDCS of some fundamental cerebral functions like attention, visuoperception, response time and working memory in healthy young health workers, are like the effects induced by diurnal napping in healthy athletes^29^. However, in fatigued and sleep-deprived health workers the potential benefits of this neuromodulatory intervention not only include the personal benefit of decrease fatal car accidents, as well decreasing the number of medical errors in hospitals to reduce morbidity and mortality^30^. For example, medication errors may account up to onethird of all medical errors in hospitals (87% prescription errors followed by administration 7.4%) where all these cognitive functions are essential^31^. Furthermore, attentional failures are associated with serious residentphysician- related medical errors, especially with shifts of 24+ hours^30^; a similar impact is observed in surgical residents with higher rates of surgical complications without any intervention. However, additional clinical trials are needed to evaluate the effects of a-tDCS in the performance of health workers in clinical settings^32^.

Remarkable, we did not find any significant effect of a-tDCS in the symptoms of anxiety-related to acute sleep deprivation in our subjects, a similar lack of effect has been reported in a new large-scale anxiety study using a single session of a-tDCS (20minx2mA) over the left dorsolateral prefrontal cortex, in which the researchers recommend stimulate other cerebral regions to obtain effects^33^.

The mechanism of action of tDCS is partially understood. Pre-clinical and clinical studies have provided evidence concerning the mechanisms underlying the acute and long-term effects of tDCS^34,35,36,37^. Original works found that weak, direct electric currents could be effectively delivered trans-cranially to induce bidirectional, polaritydependent changes in cortical neurons. Particularly, anodal stimulation was shown to increase cortical excitability, whereas cathodal stimulation decreased it^38,39^. Though, it is well known that tDCS has a partial non-linear effect depending on the strength and duration of the stimulation^40^. The acute properties are due to the primary polarization mechanism that induces changes in ionic concentrations (Na+, Ca+, K+, Cl-), alteration of the pH balance, and transmembrane protein variations by synaptic^40^ and non-synaptic mechanisms^41^. Active tDCS can induce changes in both local (ie, brain regions under the transcranial electrodes) and diffuse (ie, brain regions remote to the electrodes) regions considering the brain as an anatomic and functional complex neuronal network^42^.

A study in healthy volunteers where a-tDCS was applied on three consecutive days over the left prefrontal region showed anodal left prefrontal stimulation increased the activity of the locus coeruleus (LC)^43^; activation of LC neurons drives wakefulness and modulates associated behaviors such as attention, memory, performance, stress, and anxiety^44^. Additionally, a fMRI study that included healthy volunteers with 24 hours of acute sleep deprivation underwent a-tDCS over right dorsolateral prefrontal cortex (1.0 mA, 20 min) showed a higher and efficient functional connectivity of the thalamus with the temporal lobe and left caudate that contribute to explain the improve in cognitive performance after a-tDCS^45^. These neuroimaging findings partially explain our clinical results using a-tDCS in healthy volunteers, also in patients with fibromyalgia^46^ with acute sleep deprivation or patients with idiopathic hypersomnia^47^.

Our results post-tDCS obtained from BANFE-2 tests showed a decrease control in the orbito-medial region of the frontal lobes associated with acute sleep deprivation in the participants. External stimuli (visual, sensation and olfactory) as well as internal stimuli (visceral, gustatory, somatosensorial) all converge in this region, thus, when affected, clinical manifestations can be personality changes, cognitive dysfunction (attention, learning and working memory), and externalized behaviors. Previous neuroimaging studies found that reward-relevant brain regions that are affected by sleep deprivation include the medial prefrontal cortex, insula, orbitofrontal cortex and the striatum^48^.

Previous sleep a-tDCS clinical studies reported that the effects on brain rhythms are dependent on the brain state at the time of the therapy (wakefulness/sleep) as well as on stimulation parameters (e.g. current strength), size of electrodes, or montage used, and explain the variability of the clinical study results reported^49^. For example, a-tDCS applied bilaterally over the dorsolateral prefrontal cortex during wakefulness in healthy subjects with eyes-closed or eyes-open increase focal EEG theta power^49,50^. During sleep anodal slow-oscillatory transcranial direct current stimulation (so-tDCS) showed no effects in healthy young subjects or insomnia patients^51,52,53^. However, during slow wave sleep stages in healthy participants the findings are inconclusive with positive and negative effects in declarative and visuospatial memory^54,55^.

The adverse effects reported by the subjects in our study were mild and self-limiting, similar to those reported in other clinical studies of tDCS^56^. The most frequent were mild headache and paresthesia’s at the site of electrode placement.

The extrapolation of our findings could have ethical and administrative issues, as medical residents with long-hours of sleep deprivation have higher rates of attentional failures^57^. Current recommendations from the Accreditation Council for Graduate Medical Education (ACGME) and the Institute of Medicine (IOM) of the United States endorse a maximum that interns’ shifts not exceed 24 hours and that residents working up to 30 hours be allotted 5 hours for a nap, however, 4 to 6 additional duty hours are allowed. It is suggested to implement programs to encourage residents to use “alertness management strategies”, including napping^58,59^.

The limitations of our study include a small sample size and lack of neuroimaging or EEG to correlate with the neuro-psychological test results. Further, the lack of a control such as placebo or sham stimulation does not exclude the possibility that our findings are merely the result of the sensation of electrical stimulation, rather than more focused brain modulation. However, previous descriptive studies in medical residents with acute and chronic sleep deprivation reported an enhanced number of human error-related accidents, increased morbimortality, and a global decline in social, financial, and human productivity^4,60^.Based in these consequences, is recommended to included countermeasures as design work schedules or fatigue management such as use of caffeine and naps, none of these was observed or applied in our medical residents according to the long hours of acute deprivation mentioned and our exclusion criteria used^60^. Future studies with larger samples size, complex study design and other a-tDCS protocols and concurrent interventions such as caffeine or other CNS stimulating drugs or therapies is encouraged.

## 5. CONCLUSIONS

In summary, a- tDCS is a safe, non-invasive therapy that can improve working memory, attention and response time and distractors elimination in acute sleep deprived medical residents.
